# Impact of the COVID-19 Pandemic on the Diagnosis of Tuberculosis in Brazil: Is the WHO End TB Strategy at Risk?

**DOI:** 10.3389/fphar.2022.891711

**Published:** 2022-06-29

**Authors:** Mariana do Rosário Souza, Wandklebson Silva da Paz, Vinícius Barbosa dos Santos Sales, Gleidson Felipe Hilario de Jesus, Débora dos Santos Tavares, Shirley V. M. Almeida Lima, Álvaro Francisco Lopes Sousa, Enaldo Vieira de Melo, Rodrigo Feliciano do Carmo, Carlos Dornels Freire de Souza, Márcio Bezerra-Santos

**Affiliations:** ^1^ Health Science Graduate Program, Universidade Federal de Sergipe, São Cristóvão, Brazil; ^2^ Parasitic Biology Graduate Program, Universidade Federal de Sergipe, São Cristóvão, Brazil; ^3^ Tropical Medicine Graduate Program, Universidade Federal de Pernambuco, Recife, Brazil; ^4^ Department of Medicine, Universidade Federal de Sergipe, Aracaju, Brazil; ^5^ Department of Health Education, Universidade Federal de Sergipe, Lagarto, Brasil; ^6^ Department of Nursing, Universidade Federal de Sergipe, Lagarto, Brasil; ^7^ Global Health and Tropical Medicine (GHTM), Instituto de Higiene e Medicina Tropical, Universidade Nova de Lisboa, Lisbon, Portugal; ^8^ College of Pharmaceutical Sciences, Federal University of Vale do São Francisco (UNIVASF), Petrolina, Brazil; ^9^ Department of Medicine, Universidade Federal de Alagoas, Arapiraca, Brazil; ^10^ Department of Morphology, Universidade Federal de Sergipe, São Cristóvão, Brazil; ^11^ Laboratory of Immunology and Molecular Biology, University Hospital, Universidade Federal de Sergipe, Aracaju, Brazil

**Keywords:** tuberculosis, COVID-19, COVID-19 pandemic, end TB strategy, global healh

## Abstract

**Background:** In 2014, the World Health Organization (WHO) launched the “post-2015 End TB strategy”, that aims to end the global tuberculosis (TB) epidemic by 2030. However, the COVID-19 pandemic has severely impacted global public health and the strict measures to control the coronavirus spread can affect the management of other diseases, such as TB. Herein, we aimed to assess the impact of the COVID-19 pandemic on the diagnosis of TB in Brazil, during 2020.

**Methods:** We carried out an ecological and population-based study, using spatial analysis techniques. The variables used were the new cases of TB, pulmonary tuberculosis (PTB), and also baciloscopy-positive (BP) cases in Brazil between 2015 and 2020. The percentage of changes (% change) was calculated to verify if there was an increase or decrease of TB cases in 2020, along with time trend analyses given by Joinpoint regression model. Also, interrupted time series analyses were used to assess the trend of TB diagnosis before and after the onset of the COVID-19 in Brazil. Spatial distribution maps were elaborated, considering the % change of each Brazilian state.

**Findings:** Data analyses showed a reduction in the diagnosis of TB (−8.3%) and PTB (−8.1%) in Brazil after the irruption of the COVID-19 pandemic. Likewise, 22 states depicted a reduction in TB diagnosis. An expressive reduction of BP cases (−17.1%) was also observed. Interestingly, interrupted time series analysis showed decline in TB and PTB diagnoses from March 2020. Spatial analyses revealed that all states had a progressive reduction of TB, PTB and PB cases, from March on, with the highest percentages of reduction in December (−100% to −75%).

**Interpretation:** Taken together, our analyses demonstrated a reduction in TB diagnosis after the irruption of the COVID-19 pandemic in Brazil and its regions, signaling a serious impact on the WHO “End TB Strategy” global plan.

## Introduction

The world is currently facing major public health challenges due to the pandemic caused by the new severe acute respiratory syndrome coronavirus 2 (SARS-CoV-2) (WHO. 2020; WHO, 2021a). The first cases were reported in December 2019, in the city of Wuhan, China (Wang et al., 2020). In March 2020, the World Health Organization (WHO) declared the COVID-19 pandemic and led to the implementation of extraordinary public health measures to reduce the spread of the virus around the world (WHO, 2021b). Despite all measures recommended by the WHO, Brazil was not successful and, until May 2022, is the third in the world ranking of COVID-19 cases (about 30 millions) and second in COVID-19-related deaths (about 660 thousand) (Brasil, 2020). Additionally, the pandemic has been causing severe social, economic, and health services impacts, which can greatly affect the controlling measures of other diseases, especially those neglected, such as tuberculosis (TB).

TB is a severe infectious disease, with a chronic clinical course, caused by the intracellular bacillus *Mycobacterium tuberculosis* (Global, 2019)*.* Similar to SARS-CoV-2, the disease is also transmitted through the air, and although it may manifest in different organs or tissues, the bacillus most often affects the lungs, causing the pulmonary tuberculosis (PTB) (Matteelli et al., 2018). Importantly, TB is the leading cause of death among people living with HIV (0.4 million associated deaths) and also a major contributor to antimicrobial resistance (World Health Organization, 2017). Despite having a worldwide distribution, TB mostly affects the poorest, most vulnerable, socially marginalized, immunosuppressed, and the inequitably served by public services, especially in low- and middle-income countries (World Health Organization, 2017; Matteelli et al., 2018; Global, 2019).

TB is a constant threat to public health and according to WHO estimates, approximately 10 million new TB cases are reported worldwide annually (Global, 2019). Regardless of being a preventable and curable disease, around 1.5 million people still die from TB every year, and, before COVID-19, it was the leading infectious cause of death in the world (Global, 2019). Hereupon, WHO recommends the early detection of TB and its timely treatment, in order to reduce the risk of clinical complications, related deaths, and also the bacillus transmission (Global, 2019). More importantly, in 2014, WHO, along with the World Health Assembly, launched the “post-2015 End TB strategy”, a bold proposal for a change in coping with this disease, that aims to end the global TB epidemic by 2030. It serves as a strategic plan model for countries to reduce TB incidence by 80%, TB-related deaths by 90%, and to eliminate the high costs for TB-affected households (World Health Organization, 2017). Also, this strategy is builds on underpinned four key principles: 1) government stewardship and accountability, with monitoring and evaluation; 2) strong coalition with civil society organizations and communities; 3) protection and promotion of human rights, ethics and equity; and 4) Adaptation of the strategy and targets at country level, with global collaboration (World Health Organization, 2017).

Brazil is among the 22 countries with a high TB burden (Cortez et al., 2021). In 2017, the Brazilian Ministry of Health released the National Plan to end TB as a public health problem. This plan aimed a TB incidence rate of less than 10 cases and 1 death per 100,000 inhabitants by 2035 (Brasil M da SS de V em S, 2017). Nevertheless, new cases detection rates remain alarming; in 2019, the incidence rate was 36.6/100,000 inhabitants (Brasil M da SS de V em S, 2017; Brasil MSD de D de CC e, 2020; Cortez et al., 2021).

The COVID-19 pandemic has led almost all countries to implement unprecedented public health measures (WHO - World Health Organization, 2020a). As a result, state police actions on social distancing reduced urban mobility, and therefore limited access to health services during the pandemic, that most likely affected the diagnosis, treatment, follow-up, and control of many diseases, especially the neglected ones (WHO, 2020; Kura et al., 2021), such as leprosy (Silva da Paz et al., 2022) and hepatitis C (do Carmo and de Souza, 2022), as previously reported by our group. Recent studies have already showed a reduction on TB patient care, mainly associated with fear of SARS-CoV-2 exposure (Migliori et al., 2020; Visca et al., 2021). Thereupon, the Pan American Health Organization (PAHO) reinforced the need to maintain TB services, even in extraordinary situations, as pandemics (OPAS, 2021). In light of the above, this study aimed to evaluate the impact of the COVID-19 pandemic on TB diagnosis in Brazil during 2020.

## Materials and Methods

### Study Type and Design

An ecological and population-based study was conducted using temporal and spatial analysis techniques, using the following data: new cases of TB, and pulmonary tuberculosis (PTB), and baciloscopy-positive (BP) cases, representing Alcohol-Acid Resistant Bacillus (AARB+), in Brazil, between 1 January 2015, and 31 December 2020. The period from 2015 to 2019 was used to obtain the expected values of the events analyzed, and the year 2020 was used for comparison purposes. The expected number of cases for 2020 was assessed by calculating the average TB cases during the last 5 years prior to the pandemic (2015–2019) and compared to those detected in 2020. Herewith, it was possible to evaluate the influence of the COVID-19 pandemic on the notification of TB in Brazil during 2020.

### Data Source

Data referring to TB, PTB and baciloscopy-positive cases were collected from the Notifiable Diseases Information System (SINAN), an open access data platform of the Brazilian Ministry of Health: (http://portalsinan.saude.gov.br/dados-epidemiologicos-sinan). SINAN uses standardized forms by the Brazilian government to collect information on compulsory notifiable diseases as TB—this forms does not include personal information such as name and address (Brasil M da SS de V em S, 2019). Importantly, all TB cases are registered in the referred information system and the data on dispensing of anti-TB drugs is done exclusively by professionals of health services linked to the Brazilian federal government (Brasil MSD de D de CC e, 2020). Likewise, data on COVID-19 were extracted from the website of the Brazilian Ministry of Health launched to share COVID-19 indicators of the country with the general population (Brasil M da SPI de V em S (IVIS), 2021). Also, the digital cartographic mesh of Brazil (divided by regions and federative units or states) was extracted from the Geographical Projection System, from the website of the Brazilian Institute of Geography and Statistics (IBGE), in shapefile format (Geodetic Reference System, SIRGAS/2000).

### Study Area and Population

Brazil (Supplementary Material S1) is located in South America and has a great territorial extension of about 8,516,000 km^2^. The units of analysis were the 26 Federative Units (FU) and one Federal District, comprising 5,570 municipalities. Additionally, the FU are grouped into five geographic regions (North, Northeast, South, Southeast, and Central-West). The estimated population was 211,755,692 inhabitants for the year 2020 (IBGE, 2021). Notably, Brazil is 12th largest economy of the world, with an estimated gross domestic product (GDP) of US$ 1.434 trillion for 2021. Notwithstanding, the country exhibits serious social inequalities, as people living in extreme poverty, illiteracy, household clusters, and areas with no access to safe-drinking water and sewage system, mainly in slums (IBGE, 2021). Hence, Brazil is endemic for many neglected diseases as Chagas disease (Góes et al., 2020), leprosy (Souza et al., 2020; Damasceno et al., 2021), leishmaniasis (Ribeiro et al., 2021), schistosomiasis (Paz et al., 2020; Silva da Paz et al., 2021), and TB (Lima et al., 2019).

### Percentage of Change Calculation and Data Analysis

To assess the impact of the pandemic on TB cases reported in Brazil during 2020, the percentage of change (% change) was calculated based on the following variables: 1) new TB cases; 2) new PTB cases; 3) baciloscopy-positive cases.

The % change was primarily designed to assess disparities in the mortality rate of different health problems (Giattino et al., 2021). However, it has also been used to analyze morbidity rates (Silva da Paz et al., 2022; do Carmo and de Souza, 2022). Considering the expected value and the one observed, it is possible to calculate the increase or reduction in the phenomenon occurrence in time and space (Giattino et al., 2021). Therefore, an adaptation of the % change approach was applied, as follows:

$$\%\;change\;\;\;=\frac{\;Number\;of\;TB\;cases\;registered\;in\;2020-number\;of\;TB\;cases\;\exp ected\;in\;2020\;}{Number\;of\;TB\;cases\;\exp ected\;in\;2020}x\;100$$

in which, the number of cases registered in 2020 corresponds to the official data monthly notified by the Brazilian Ministry of Health; and the number of cases expected for 2020 corresponds to the average of cases registered monthly in the 5 years prior to the pandemic year (2015–2019), as recommended (Giattino et al., 2021).

The results are expressed as percentages: 1) positive values indicate an increase; 2) and negative values indicate a decrease in the number of cases compared to the expected values (Giattino et al., 2021). The % change was assessed by regions, states and the entire country. Results were presented as bar graphs and timelines showing both observed and expected values for TB and COVID-19 monthly indicators in 2020. Microsoft Office Excel^®^ software 2017 (©Microsoft) was used for % change analyses and graphs elaboration.

### Temporal Trend Analyses

A segmented log-linear regression, using the joinpoint regression model, was used to assess the temporal trend of tuberculosis according to the following variables: 1) new TB cases; 2) new PTB cases; and 3) baciloscopy-positive cases in Brazil and its regions, between 2015 and 2020. The Monte Carlo permutation test was applied to select the best model for inflection points (applying 999 permutations), considering the highest residue determination coefficient (R2). Firstly, a temporal trend of all variables between 2015 and 2019 was elaborated, to verify the behavior of TB in Brazil and its regions before the COVID-19 pandemic. Afterwards, the data of the year 2020 was added and a new time trend analysis was done to identify the impact of the pandemic on TB diagnosis. Furthermore, to describe the temporal trends, we calculated the monthly percentage change (MPC) and its respective confidence interval (CI 95%) (Institute, 2013). Time trends were considered statistically significant when MCP had a *p*-value < 0.05 and their CI 95% did not include zero. Importantly, a positive and significant MCP values indicate an increasing trend; alternatively, a negative and significant MPC indicates a decreasing trend; and non-significant trends are described as stable, regardless of MPC values (Institute, 2013).

### Interrupted Time Series Analyses

We conducted an interrupted time series analyses to assess whether the TB diagnosis in 2020 in Brazil, after the irruption of the COVID-19, differ from the trend between 2015 and 2019. The variables evaluated were the monthly detection of TB, PTB, and baciloscopy-positive. The intervention model was the onset of the COVID-19 pandemic in Brazil in March 2020. First, the graphs of residue and sample and partial autocorrelation function (ACF and partial ACF) were used to identify autocorrelation in the residue and properties of stationarity and normality, to select the most appropriate and statistically parsimonious models (Oliveira et al., 2019). Thereafter, the ARIMA models of serial dependence were determined. The selected preintervention model was an ARIMA 2,1,0. Lastly, the Ljung-Box (Q) test was applied to verify whether the residuals were white noise (approximately normally distributed around zero) (Aditya Satrio et al., 2021). Importantly, the Ljung-Box test indicated that the models are applicable to describe the linear dependence among successive repetitions. These analyses were carried out using IBM SPSS Statistics software version 22.

### Spatial Analysis of Data and Preparation of Choropleth Maps

Choropleth maps showing the spatial distribution of the % change values per state, during the months of 2020, were elaborated. The cartographic base of Brazil, divided by regions and states, made available by the IBGE on address https://portaldemapas.ibge.gov.br/portal.php#homepage, was used.

The following parameters were used to create the maps: 1) the % change related to new TB cases per month; 2) % change in the number of PTB cases per month; 3) and % change in the number of baciloscopy-positive cases per month. Furthermore, choropleth maps were stratified into nine categories of equal intervals, according to P-score (positive or negative) percentages: −100 to −75%; −75 to 50%; −50 to −25%; −25 to −0.1%; 0%; 0.1 to 25%; 25 to 50%; 50 to 75%; >75%. QGis software version 3.18.3 (QGIS Development Team; Open-Source Geospatial Foundation Project) was used to prepare the maps.

## Results

**FIGURE 1:**
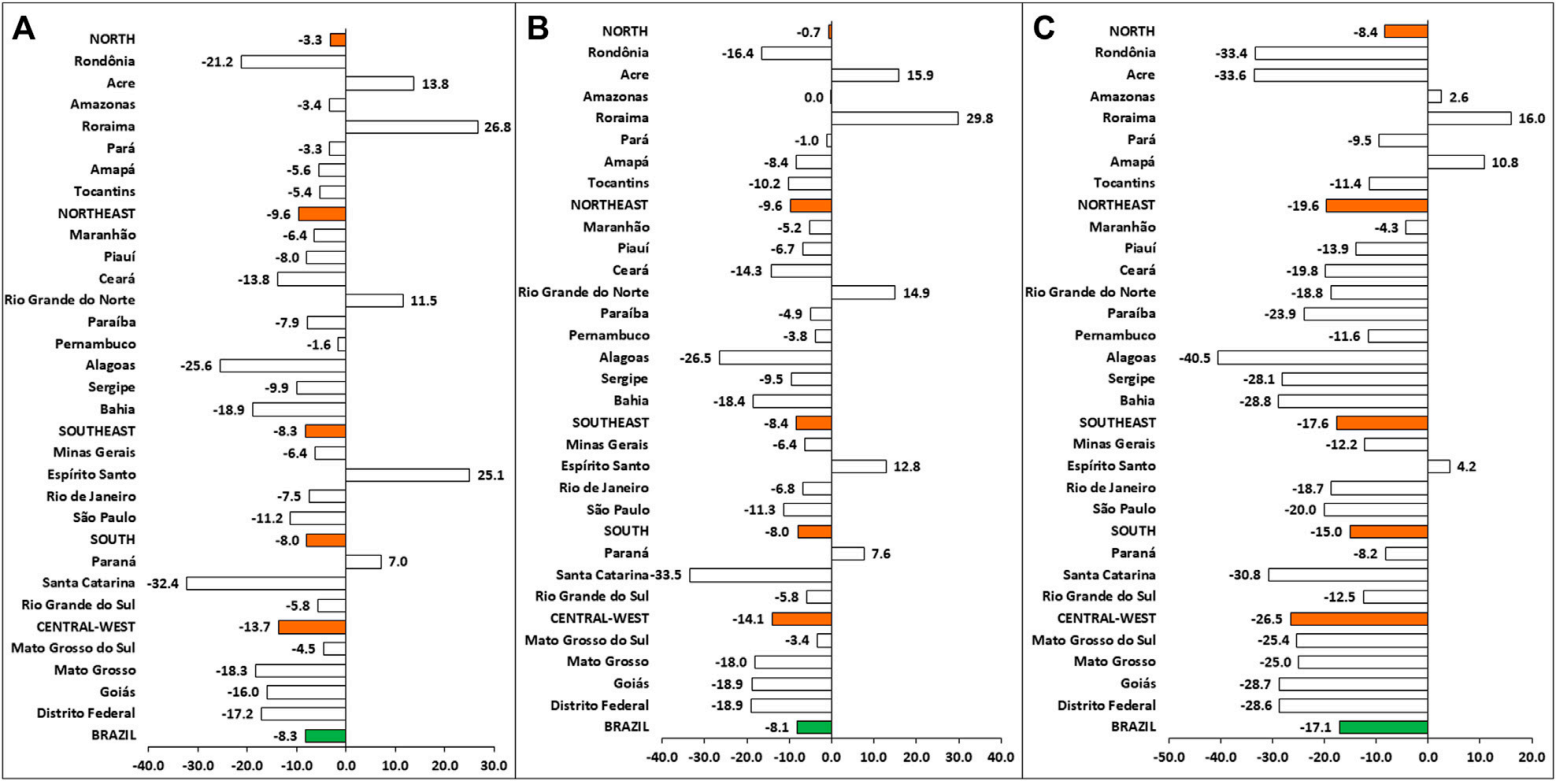
The percentage of change of tuberculosis cases, according to data from Brazil (green bars), Brazilian regions (orange bars), and states. **(A)** % change in the new TB cases; **(B)** % change in the new PTB cases; **(C)** % change in the new baciloscopy-positive (AARB+) cases.

In Brazil, 83,678 cases of TB were reported in 2020. Nevertheless, according to the average number of cases in the last 5 years (2015–2019), approximately 91,225 cases were expected in 2020, depicting a reduction of 7,547 cases (% change: −8.3%) (Figure 1A). Likewise, all Brazilian regions exhibited a negative % change in TB cases. After analyzing all Brazilian states, 22 showed a reduction in the diagnosis of TB in 2020; the highest negative percentages were observed in Santa Catarina (−32.4%), Alagoas (−25.6%), and Rondônia (−21.2%). Conversely, five states showed an increase in the number of diagnoses, especially Roraima (26.8%) and Espírito Santo (25.1%).

As for pulmonary tuberculosis, 70,855 cases were reported in 2020, in contrast to the 77,090 estimated cases for this period (reduction of 6,235 PTB cases; −8.1%) (Figure 1B). Similarly, all regions presented a negative % change, especially the Central-West region (−14.1%). After analyzing state’s % change, it was verified a reduction in PTB cases of 22 states, showing values similar to those observed for TB cases.

Interestingly, a marked reduction in the number of baciloscopy-positive (AARB+) cases was noted in Brazil during 2020. Considering the average of the last 5 years (2015–2019), it was estimated 45,898 AARB+ cases. However, 38,027 AARB+ were reported in SINAN, representing a reduction of 7,871 AARB+ cases (−17.1%) (Figure 1C). Likewise, there was a reduction in all Brazilian regions, especially in the Central-West (−26.5%), Northeast (−19.6%) and Southeast (−17.6%) regions. Remarkably, 15 states showed percentage reductions higher than the national average, especially Alagoas (−40.5%), Acre (−33.6%), Rondônia (−33.4%), and Santa Catarina (−30.8%).

From March to December 2020, the % change curve showed a progressive reduction in monthly notifications of new TB cases in all Brazilian regions after the beginning of the pandemic in the country (Supplementary Material S2A–E). The North, Northeast, and Central-West regions exhibited the highest negative % change in December (−49.4%, −35.8%, and −21.8%, respectively). As expected, this reduction was also observed in the PTB cases (Supplementary Material S3A–E). The highest negative % change occurred in December, mainly in the North, Northeast, and Central-West regions (−48.1%, −35.5%, and −73.7%, respectively).

Likewise, there was a reduction in the number of baciloscopy-positive cases in all Brazilian regions (Supplementary Material S4A–E). From March on, after the first confirmed COVID-19 case in Brazil, the decrease of positive cases was more pronounced, both nationally and regionally, with the highest reductions observed in December, especially in the North and Central-West regions (−52.5% and −69.8%, respectively).

**FIGURE 2:**
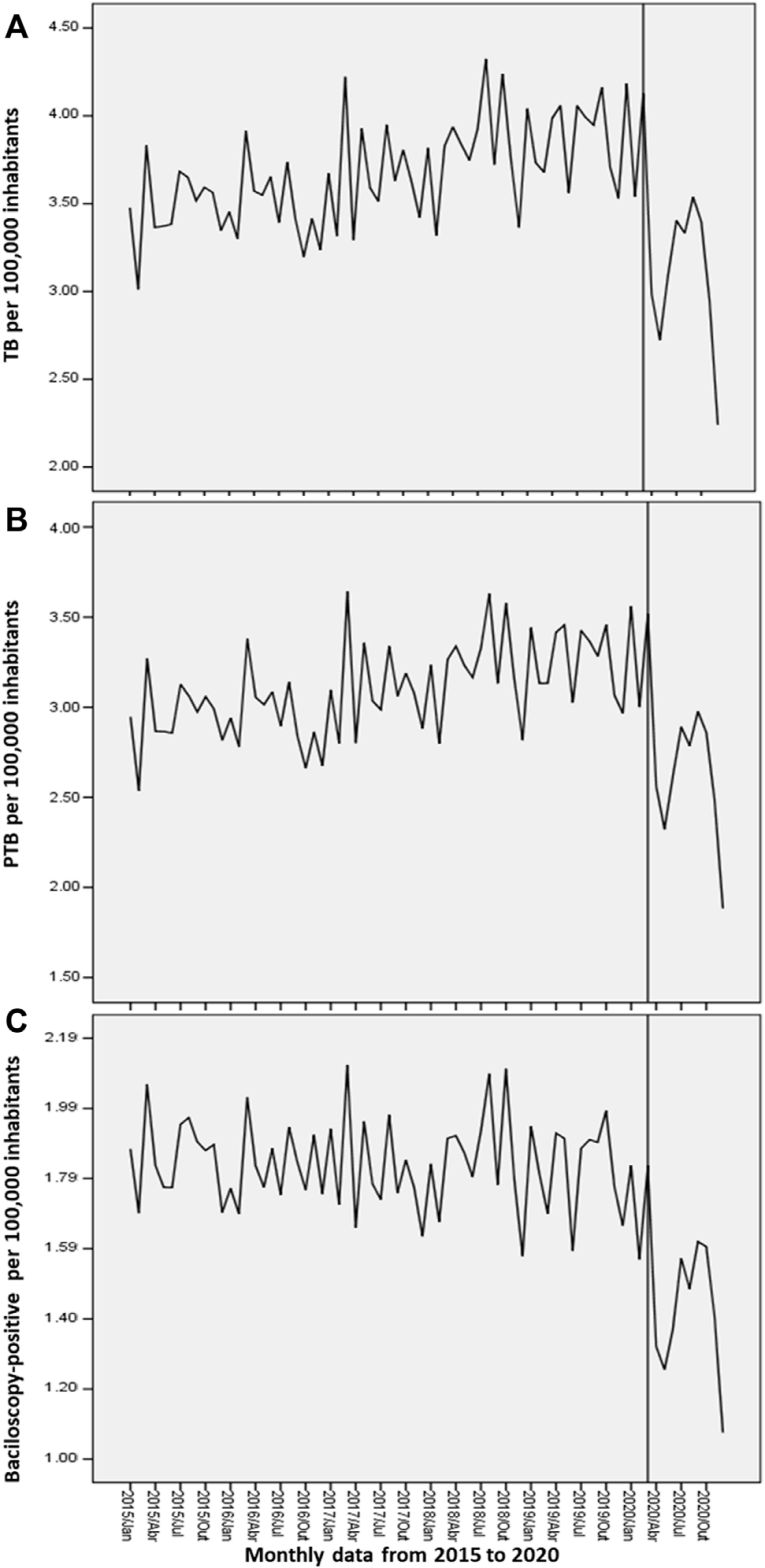
Interrupted time series analysis of tuberculosis cases in Brazil: **(A)** New cases of tuberculosis (TB); **(B)** New cases of pulmonary tuberculosis (PTB); **(C)** Baciloscopy-positive (AARB+) cases. The line that cuts each time series indicates the intervention in the series, in this case, the onset of the COVID-19 pandemic in Brazil in March 2020.

The coefficient for the detection of TB and PTB (per 100,000 inhabitants) increased from 2015 to 2019 in Brazil and all the regions. Impressively, all coefficients in 2020 were lower than the ones reported in 2015 (Table 1 **).** Besides, time trend analyses in the years prior to the COVID-19 pandemic (2015–2019) confirmed increasing trends in TB and PTB diagnosis in Brazil (APC: 3.8% and 3.7%, respectively) and across all regions. The North, Northeast and South regions had the highest MPC. Regarding the baciloscopy-positive cases, there was a stable trend in Brazil, but an increasing trend in the North and Central-West regions. Subsequently, the interrupted time series analysis was applied to verify whether the irruption of the COVID-19 affected the diagnosis of TB in Brazil in 2020. Corroborating the joinpoint analyses, we observed a not stationary and a decreasing trend in the diagnosis of TB in Brazil (stationary R^2^ = 0.578; normalized BIC = −2.19; significance = 0.019; ARIMA estimate = −0.081; *p*-value = 0.06; Figure 2A), after the establishment of the pandemic in March 2020. Likewise, there was a not stationary and decreasing trend in the diagnosis of PTB (stationary R^2^ = 0.573; normalized BIC = −2.436; significance = 0.014; ARIMA estimate = −0.073; *p*-value = 0.066; Figure 2B) and baciloscopy-positive (stationary R^2^ = 0.388; normalized BIC = −3.357; significance = 0.008; ARIMA estimate = −0.043; *p*-value = 0.14; Figure 2C) from March 2020.

**TABLE 1:** Temporal trends of tuberculosis detection coefficient (per 100,000 inhabitants) in Brazil and its regions between 2015 and 2020.

Variables/areas	2015	2019	MPC	CI 95%	Trend	2020	MPC	CI95%	Trend
Pulmonary and extrapulmonary tuberculosis (TB)									
Brazil	41.80	46.46	3.8*	2.3; 5.2	Increasing	39.52	0.9	−3.6; 5.6	Stable
North	51.58	63.66	6.6*	3.9; 9.3	Increasing	52.52	3.3	−2.0; 8.8	Stable
Northeast	39.25	45.11	4.8*	2.1; 6.7	Increasing	37.97	1.0	−4.2; 6.6	Stable
Southeast	45.71	48.39	2.6*	1.1; 4.0	Increasing	42.40	0.2	−3.5; 4.1	Stable
South	37.72	42.67	4.3*	0.8; 7.9	Increasing	35.27	1.2	−3.8; 6.5	Stable
Central-West	26.02	28.19	3.9*	1.3; 6.5	Increasing	22.38	0.1	−5.8; 6.4	Stable
Pulmonary tuberculosis (PTB)									
Brazil	35.37	39.17	3.7*	2.2; 5.2	Increasing	33.46	0.9	−3.5; 5.5	Stable
North	44.08	54.76	6.7*	3.8; 9.6	Increasing	46.32	3.7	−1.1; 8.8	Stable
Northeast	33.93	38.39	4.0*	1.5; 6.6	Increasing	32.52	0.8	−4.3; 6.2	Stable
Southeast	38.51	40.62	2.5*	1.0; 4.0	Increasing	35.60	0.2	−3.6; 4.0	Stable
South	30.57	34.98	4.6*	1.8; 8.0	Increasing	28.82	1.4	−3.8; 6.9	Stable
Central-West	22.43	24.15	3.6*	1.3; 5.9	Increasing	19.14	−0.1	−6.0; 6.0	Stable
Baciloscopy-positive (AARB+)									
Brazil	22.36	22.04	0.5	−0.4; 1.4	Stable	17.96	−2.4	2.3; −1.4	Stable
North	27.09	31.65	4.6*	1.7; 7.6	Increasing	26.01	1.4	−3.9; 6.9	Stable
Northeast	21.82	21.66	0.7	−1.8; 3.3	Stable	17.38	−2.7	−8.0; 3.0	Stable
Southeast	24.39	22.14	−1.6*	−2.1; −1.0	Decreasing	18.71	−3.6*	−6.8; −0.3	Decreasing
South	19.95	21.20	2.8	−0.3; 6.0	Stable	16.85	−0.7	−6.4; 5.3	Stable
Central-West	12.25	13.45	3.8*	0.5; 7.2	Increasing	8.82	−2.2	−11.3; 7.8	Stable

MPC, monthly percentage change; CI, confidence interval; *p-value < 0.05; AARB+, Alcohol-Acid Resistant Bacillus.

**FIGURE 3:**
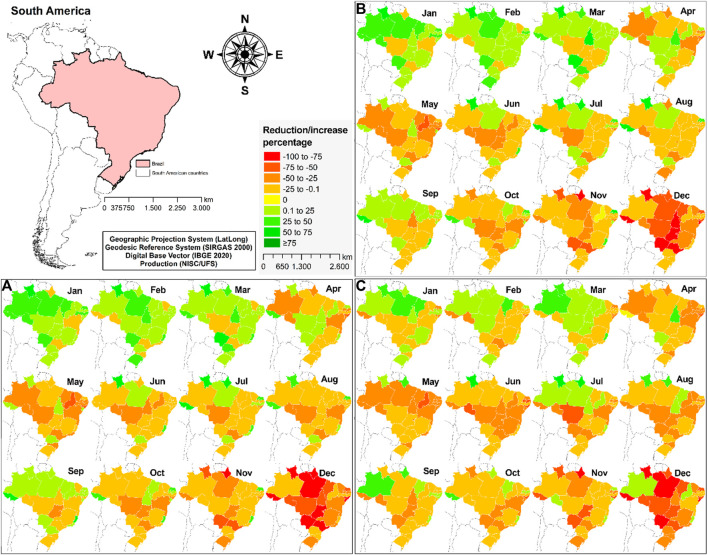
Spatial distribution of the monthly percentage of change in the TB cases in Brazil, between January and December 2020: **(A)** New cases of tuberculosis (TB); **(B)** New cases of pulmonary tuberculosis (PTB); **(C)** Baciloscopy-positive (AARB+) cases.

Regarding the spatial distribution of new TB cases (Figure 3A), from March on, all states had a progressive reduction in the number of cases. More importantly, nine states had the highest percentages of reduction in December (−100% to −75%, in red). Similar patterns were observed for PTB (Figure 3B), as most states presented a reduction in PTB cases after March. In addition, eight states had the highest reduction in December (−100% to −75%, in red). Finally, as for the spatial distribution of AARB+ cases, a similar pattern was observed throughout the year of 2020 (Figure 3C). Most states (*n* = 23) showed negative % changes in December, and the highest reduction (−100% to −75%, in red) were located in five states.

## Discussion

To the best of our knowledge, this is the first study to assess the impact of the COVID-19 pandemic in the number of TB cases in Brazil. The performed analyses showed a reduction in the notifications of TB (−8.3%), PTB (−8.1%), and baciloscopy-positive (−17.1%) cases in Brazil after the onset of COVID-19 pandemic. Likewise, this reduction was also observed in all regions and most of Brazilian states. Importantly, time trend analyses showed a progressive increasing number of new TB and PTB cases in the years prior the pandemic, becoming stable in 2020. Taken together, the compiled data demonstrate the negative effect that the pandemic can cause on the WHO “End TB strategy” program in a country considered a priority for the control of the disease in the world, pointing out an insidious scenario regarding the evolution and dissemination of tuberculosis in the next years.

It is known that TB mainly affects poor and most vulnerable populations around the world, aggravating current inequalities (World Health Organization, 2020b). Despite being preventable and curable disease, before the COVID-19 pandemic TB was the main cause of death worldwide among infectious diseases, ranking alongside HIV/AIDS. Considering the great TB impact and burden to the public health worldwide, WHO launched a new global strategy (“End TB”) aiming a TB-free world by 2035. However, regardless the 47% and 42% TB mortality and prevalence rate reduction since 1990, it is still considered a serious infectious disease, mostly affecting low- and middle-income countries, as Brazil. In addition, there was a reduction in HIV and TB related deaths by 32% in the last decade (Global, 2019). Notwithstanding, time trend analyses showed increasing trends in TB and PTB diagnosis in Brazil and its regions between 2015 and 2019. These findings indicate that the epidemiological scenario of this disease in Brazil had been worsening even before the onset of the pandemic. On the other hand, this increase may be the result of improvements in TB diagnosis programs in the country. More importantly, the interrupted time series analyses corroborate the decreasing trends in the diagnosis of TB in Brazil from March 2020, highlighting the impact of the COVID-19 pandemic on tuberculosis diagnosis and control.

Notably, the irruption of the COVID-19 pandemic has caused several health problems globally, but higher impacts are seen in specific countries who had a worse coping with the disease, such as Brazil (Martins-Filho et al., 2020). Considering the pandemic effects on public health, along with the negative impact on the economy, reflected by the increased number of people living in low-income households in areas of social vulnerability, we ask the following question: Is the WHO “End TB strategy” at risk in Brazil? Our findings suggest that it does. The reduction in the TB cases during the first year of the pandemic does not express the reality. On the contrary, it is quite possible that the diagnosis of TB case numbers can be hidden by the critical COVID-19 pandemic. This scenario must be considered by health managers, since the risk of contamination and transmission of TB worldwide is already proven to be high. Also, the Brazilian situation identified in this study most likely reflects a global pattern.

The % change curve demonstrated a progressive reduction in monthly notifications of new TB and PTB cases and also baciloscopy-positive cases in all regions throughout the country after the onset of the pandemic in Brazil (from March to December 2020). TB is not a seasonal disease and can affect different age groups, so new cases are expected during the whole year and in all states of Brazil. Despite being a mandatory notifiable disease in Brazil, the obligation of immediate notification is probably being compromised by the COVID-19. As expected, the negative effects of the pandemic on the diagnosis of other diseases are being reported globally (Matos et al., 2021).

Also, due to the COVID-19 pandemic, the Brazilian health system directed its efforts to face the spread of the virus throughout the country, by building temporary hospitals and expanding the number of intensive care units (do Carmo and de Souza, 2022). Additionally, health managers have established several nonpharmacological measures, such as reducing urban mobility and limiting hours and number of daily appointments of health units. Altogether, these strategies, although necessary, may have affected the diagnosis and timely treatment of other endemic infectious diseases, as already observed in investigations involving leprosy (Silva da Paz et al., 2022) and hepatitis C (do Carmo and de Souza, 2022) in Brazil and leprosy (Matos et al., 2021) and tuberculosis (De Souza et al., 2020) in the state of Bahia (Brazil).

In this sense, time trend analyses demonstrate that TB diagnosis in Brazil was increasing until the year before the pandemic. Probably, due to the onset of the pandemic and the changes in the routine of health services, such as the relocation of health professionals to face COVID-19 and the reduction in the care provided to patients with other diseases (WHO - World Health Organization, 2020a), there was also a reduction in the TB diagnosis, reflected by the stable trends observed after adding data of the year 2020.

Herewith, those data predict a severe impact on public health. The reduction in the diagnosis of cases increases the risk of underreporting and, in consequence, a late diagnosis that may lead to disease worsening (De Souza et al., 2020) along with clinical complications. Therefore, the patient can remain for a long time with a high bacillary load, increasing the risk of *M. tuberculosis* transmission to close contacts (Global, 2019; World Health Organization, 2020b).

Surprisingly, the treatment for multidrug-resistant tuberculosis (MDR-TB) increased together with the case numbers reported in 2014 (Global, 2019). Nonetheless, MDR-TB is still considered a public health crisis. Social distancing measures, temporary closure of outpatient clinics, and the fear of SARS-CoV-2 exposure , can compromise not only TB care and diagnosis, but also the access and the adherence to treatment (De Souza et al., 2020). Importantly, treatment dropout appears as the main risk factor for MDR-TB. Thus, it is extremely necessary that health professionals provide clear information, and support to patients during the treatment to reduce the risk of abandonment. If treatment is not completed properly, the disease may become drug resistant and the resistant bacteria could spread easily (Global, 2019; World Health Organization, 2020b).

TB and COVID-19 are both airborne infectious diseases that primarily attack the lungs. They have similar symptoms such as cough, fever, and dyspnea. Hence, differential diagnosis between COVID-19 and TB should be carefully conducted, observing all clinical characteristics (Comella-del-Barrio et al., 2021), but even so laboratory testing are essential to detect co-infection cases and treat promptly to avoid severe symptoms and even death (Comella-del-Barrio et al., 2021). Furthermore, we believe that with social distancing measures and the greater number of individuals in contact in households, where people usually do not wear masks, there is an increased risk of SARS-Cov-2 transmission. This risk is even greater in urban agglomerations and areas of greater social vulnerability, such as favelas, which were mostly affected by the pandemic (Martins-Filho et al., 2020).

WHO conducted a survey among officials that worked at different Ministries of Health, located in a total of 165 countries, and reported that after the first 3 months of the COVID-19 pandemic, 42% of countries reported partial disruptions in TB case detection and treatment (Comella-del-Barrio et al., 2021). Likewise, another survey conducted in 33 centers of 16 countries on five continents reported that during confinement 82% of centers showed reductions in TB-associated hospital discharges, 84% of centers reported a decrease in recently diagnosed active TB cases , 95% showed a decrease in newly latent TB infection (LTBI) outpatient visits, and 75% and 81% of centers showed reductions in TB and LTBI outpatient visits, respectively (Migliori et al., 2020). Visca and colleagues (Visca et al., 2021) reported that 15 countries also had their essential services for TB severely hampered due to the COVID-19 pandemic.

Estimates of the impact of the COVID-19 pandemic on TB care suggest that a 3-month lockdown and a 10-month protracted recovery could result in 6.3 million additional TB cases between 2020 and 2025 (World Health Organization, 2020b). More importantly, it can cause approximately 1.4 million additional TB deaths during this period. Unfortunately, these data could imply a regression of at least 5–8 years in the fight against TB, and also put at risk the WHO “End TB strategy” plan (World Health Organization, 2020b). Additionally, Comella-del-Barrio and colleagues (Comella-del-Barrio et al., 2021) attested that a 25% reduction in global TB detection over 3 months is predicted to lead to a 13% increase in TB deaths, bringing TB mortality rates back to the numbers reported in 2015.

Importantly, the intensive use of face masks and home confinement may also have reduced TB transmission during the pandemic (Comella-del-Barrio et al., 2021). Conversely, household agglomerations imposed to reduce the spread of the SARS-CoV-2 probably increased the risk of transmission among close contacts. Therefore, it would be necessary to include these variables in predictive analyses to assess the long-term effect these measures could have on TB control.

This study has some limitations that deserve to be mentioned. First, an ecological study using secondary data was conducted, therefore, there is a risk of bias, mainly regarding to the quality of information. Additionally, TB diagnosis may be underreported in some states, or even a registration delay in the SINAN might be present. Also, data on COVID-19 may not be accurately notified, for instance there might be registration delays altering the diagnosis date.

Taken together, our analyses demonstrated that the emergence of the COVID-19 pandemic negatively impacted the diagnosis of TB in Brazil. Time trend analyses showed an increasing trend in disease diagnosis between 2015 and 2019. However, trends became stable after adding the data of 2020, the COVID-19 pandemic year. Hence, the progress made over decades of efforts to fight TB, trough the National Plan for Ending Tuberculosis, a disease considered a public health problem in Brazil, can be compromised, putting at risk the global goal of “End TB strategy”. Hereupon, public health authorities and police makers should implement emergency actions to control TB.

It is essential to train and prepare health care professionals, especially from basic health units, in order to accurate detect cases, to rapid commencement of and adherence to treatment and also to active-case finding, even in adverse times such as the pandemic. Priority should be given to the poorest populations and risk groups (people living with HIV, migrants, refugees, and prisoners), that are usually more affected by the disease. Notably, the pandemic is already showing a greater impact on areas of greater social vulnerability, and consequences can be disastrous for other diseases such as TB. In this context, global support must be provided to enable the countries and communities most affected by TB to respond to the pandemic, ensuring that TB services are maintained uninterrupted.

## Data Availability

The data sets related to tuberculosis analysed and that support the results of this study are registered in the Sistema de Informação de Agravos de Notificação (SINAN) and are available on the website of the Departamento de Informatica do Sistema Único de Saúde (DATASUS). http://tabnet.datasus.gov.br/cgi/tabcgi.exe?sinannet/cnv/hanswbr.def Data related to COVID-19 cases are also available for anyone to access without the need to request any responsible agency. http://plataforma.saude.gov.br/ For the construction of spatial analysis maps, the cartographic base of Brazil was used, available in the electronic database of the Instituto Brasileiro de Geografia e Estatística (IBGE). https://www.ibge.gov.br/geociencias/organizacao-do-territorio/malhas-territorio/15774-malhas.html?=&t=o-que-e.
