# Corrigendum: Impact of the COVID-19 pandemic on the diagnosis of *tuberculosis* in Brazil: Is the WHO end TB strategy at risk?

**DOI:** 10.3389/fphar.2022.999301

**Published:** 2022-09-06

**Authors:** Mariana do Rosário Souza, Wandklebson Silva da Paz, Vinícius Barbosa dos Santos Sales, Gleidson Felipe Hilario de Jesus, Débora dos Santos Tavares, Shirley V. M. Almeida Lima, Álvaro Francisco Lopes Sousa, Enaldo Vieira de Melo, Rodrigo Feliciano do Carmo, Carlos Dornels Freire de Souza, Márcio Bezerra-Santos

**Affiliations:** ^1^ Health Science Graduate Program, Universidade Federal de Sergipe, São Cristóvão, Brazil; ^2^ Parasitic Biology Graduate Program, Universidade Federal de Sergipe, São Cristóvão, Brazil; ^3^ Tropical Medicine Graduate Program, Universidade Federal de Pernambuco, Recife, Brazil; ^4^ Department of Medicine, Universidade Federal de Sergipe, Aracaju, Brazil; ^5^ Department of Health Education, Universidade Federal de Sergipe, Lagarto, Brasil; ^6^ Department of Nursing, Universidade Federal de Sergipe, Lagarto, Brasil; ^7^ Global Health and Tropical Medicine (GHTM), Instituto de Higiene e Medicina Tropical, Universidade Nova de Lisboa, Lisbon, Portugal; ^8^ College of Pharmaceutical Sciences, Federal University of Vale do São Francisco (UNIVASF), Petrolina, Brazil; ^9^ Department of Medicine, Universidade Federal de Alagoas, Arapiraca, Brazil; ^10^ Department of Morphology, Universidade Federal de Sergipe, São Cristóvão, Brazil; ^11^ Laboratory of Immunology and Molecular Biology, University Hospital, Universidade Federal de Sergipe, Aracaju, Brazil

**Keywords:** tuberculosis, COVID-19, COVID-19 pandemic, end TB strategy, global health

In the published article, there was an error in the Funding statement. The manuscript was published without due mention of the funding agency. The correct Funding statement appears below.

“Global Health and Tropical Medicine, Instituto de Higiene e Medicina Tropical, Universidade NOVA de Lisboa, Portugal, ref. UID/04413/2020.”

In addition, the article was published without including the acknowledgments. The correct Acknowledgments statement appears below.

“Foundation for Science and Technology (Fundação para a Ciência e tecnologia) for funds to GHTM – UID/04413/2020”

