# Supplementary figures and images for: Impact of the COVID-19 Pandemic on the Diagnosis of Tuberculosis in Brazil: Is the WHO End TB Strategy at Risk?
Source: Front Pharmacol. 2022 Jun 29;13:891711. doi: 10.3389/fphar.2022.891711 (PMC9277074; doi:10.3389/fphar.2022.891711)

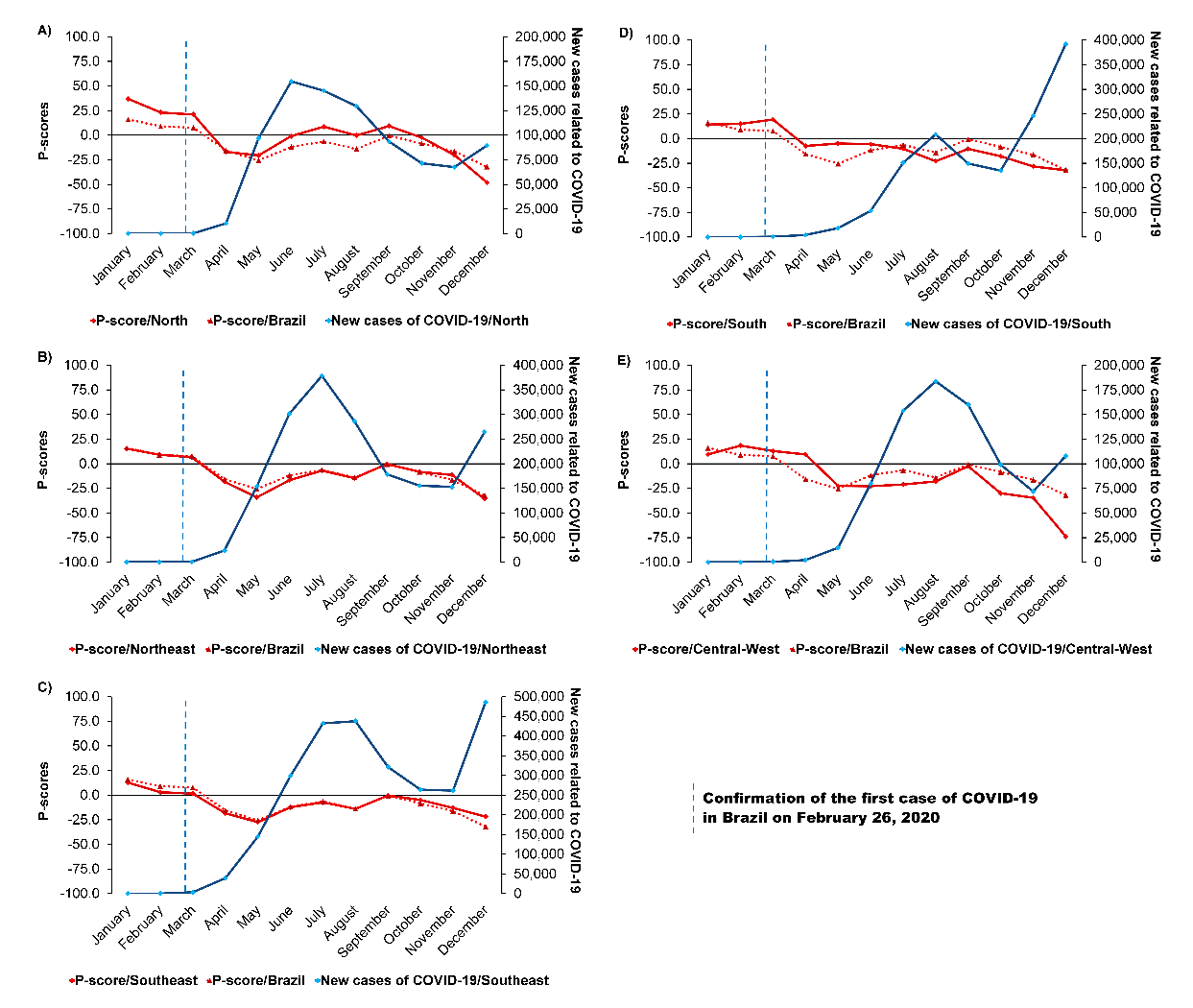

Supplement: Supplementary file 1 [file Image3.tif]

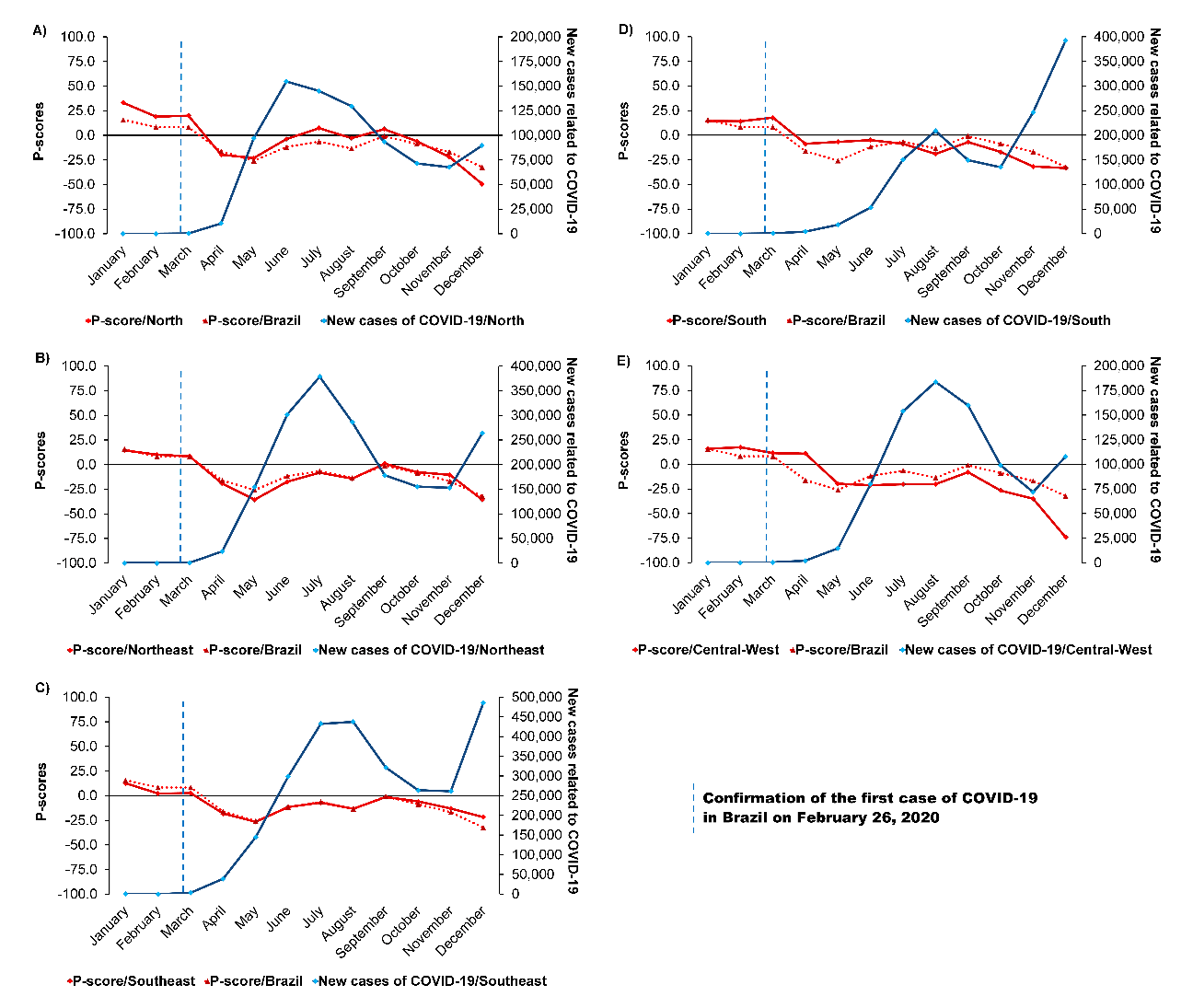

Supplement: Supplementary file 2 [file Image4.tif]

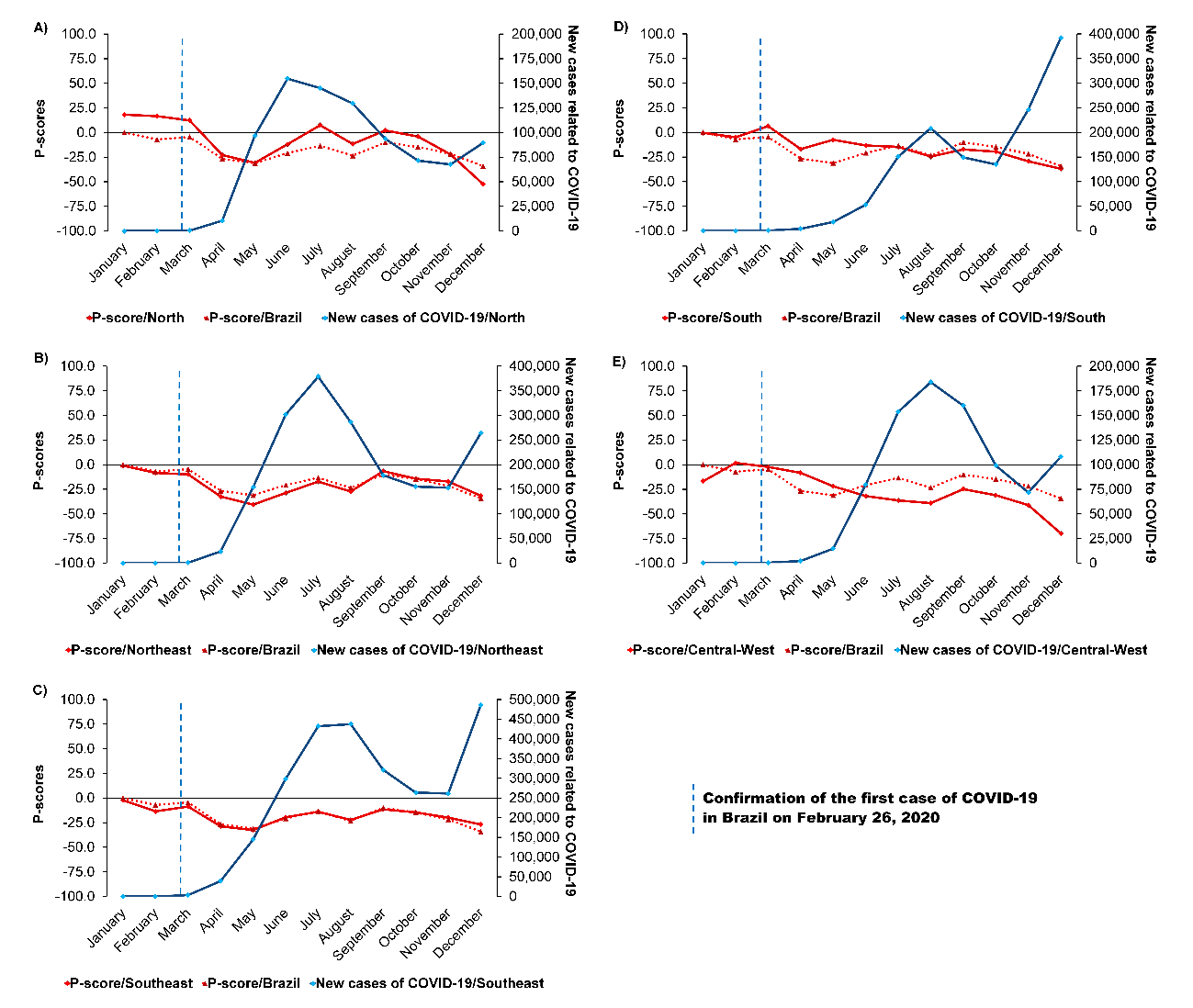

Supplement: Supplementary file 3 [file Image2.tif]

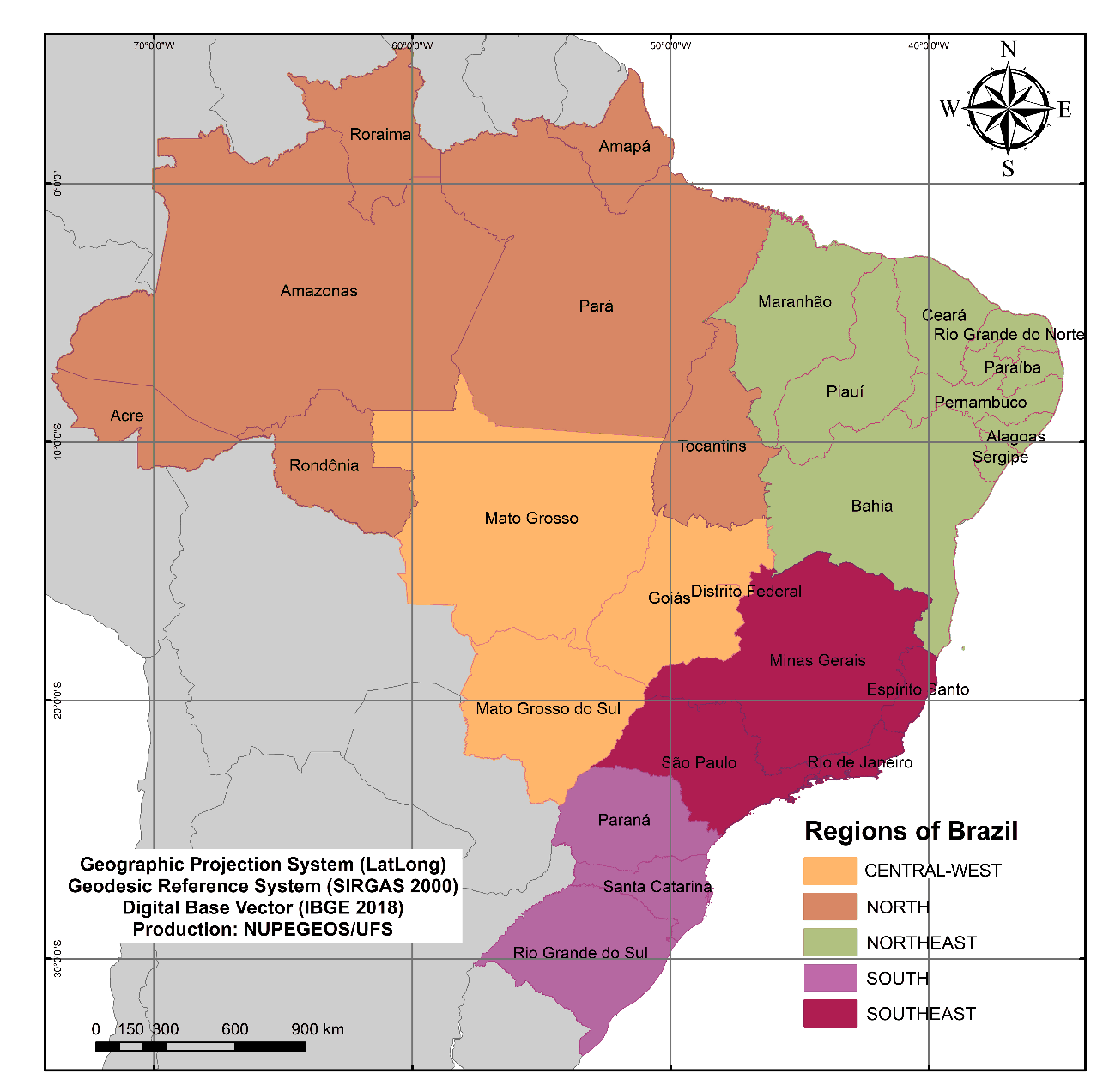

Supplement: Supplementary file 4 [file Image1.tif]
