# Supplementary material for: Impact of the COVID-19 Pandemic on the Diagnosis of Tuberculosis in Brazil: Is the WHO End TB Strategy at Risk?
Source: Front Pharmacol. 2022 Jun 29;13:891711. doi: 10.3389/fphar.2022.891711 (PMC9277074; doi:10.3389/fphar.2022.891711)
Supplement: Supplementary file 5 [file DataSheet1.docx]

**Caption for supplementary material**

**Supplementary material 1.** Map representing the study area (Brazil) divided into five geopolitical regions (Central-West, North, Northeast, South and Southeast) and 27 federative units.

**Supplementary material 2.** Monthly temporal evolution of % change in new cases of tuberculosis and COVID-19, in Brazil and its regions, between January and December 2020: **A)** North; **B)** Northeast; **C)** Southeast; **D)** Southern; **E)** Central-West.

**Supplementary material 3.** Monthly temporal evolution of % change in new cases of pulmonary tuberculosis (PTB) and COVID-19, in Brazil and its regions, between January and December 2020: **A)** North; **B)** Northeast; **C)** Southeast; **D)** Southern; **E)** Central-West..

**Supplementary material 4.** Monthly temporal evolution of % change in new baciloscopy-positive (AARB+) and COVID-19 cases, in Brazil and its regions, between January and December 2020: **A)** North; **B)** Northeast; **C)** Southeast; **D)** Southern; **E)** Central-West
